# Deep-learning-based automatic liver segmentation using computed tomography images in dogs

**DOI:** 10.3389/fvets.2025.1681820

**Published:** 2025-10-21

**Authors:** Seungyeon Lee, Genya Shimbo, Nozomu Yokoyama, Kensuke Nakamura, Ren Togo, Takahiro Ogawa, Miki Haseyama, Mitsuyoshi Takiguchi

**Affiliations:** ^1^Laboratory of Veterinary Internal Medicine, Department of Veterinary Clinical Science, Graduate School of Veterinary Medicine, Hokkaido University, Sapporo, Japan; ^2^Veterinary Teaching Hospital, Graduate School of Veterinary Medicine, Hokkaido University, Sapporo, Japan; ^3^Faculty of Information Science and Technology, Hokkaido University, Sapporo, Japan

**Keywords:** artificial intelligence, deep learning, automatic segmentation, liver, canine, dog, computed tomography

## Abstract

**Introduction:**

Deep learning-based automated segmentation has significantly improved the efficiency and accuracy of human medicine applications. However, veterinary applications, particularly canine liver segmentation, remain limited. This study aimed to develop and validate a deep learning model based on a 3D U-Net architecture for automated liver segmentation in canine abdominal computed tomography (CT) scans.

**Methods:**

A total of 221 canine abdominal CT scans were analyzed, comprising 159 cases without hepatic masses and 62 cases with hepatic masses. The model was trained and evaluated using two separate datasets: one containing cases without hepatic masses (Experiment 1) and the other combining cases with and without hepatic masses (Experiment 2).

**Results:**

Both experiments demonstrated high segmentation performance, achieving mean Dice similarity coefficients of 0.926 (Experiment 1) and 0.929 (Experiment 2).

**Discussion:**

The manual and predicted liver volumes showed excellent agreement, highlighting the potential clinical applicability of this approach.

## 1 Introduction

Medical image segmentation, the process of delineating a medical image into distinct anatomical structures, is an essential technology in diagnostic imaging that enables clinicians to precisely analyze complex structures. In human medicine, deep-learning-based segmentation techniques have greatly improved clinical workflows by enhancing diagnostic accuracy and efficiency (1–3). These advancements support clinical applications, such as surgical planning, radiation therapy, disease monitoring, and medical education (4). Among the various organs, the liver is a key target for segmentation because of its complex anatomy and role in numerous diseases. Accurate segmentation of liver structures facilitates quantification tasks such as liver volumetry, which are essential for clinical decision-making, including surgical resections and disease management (5–7). Recent deep learning-based segmentation models have demonstrated the capability to accurately segment liver structures from computed tomography (CT) images, achieving performance comparable to that of expert manual segmentation (8, 9).

However, in veterinary medicine, segmentation techniques are still in their early stages of development (10, 11). Current practice relies primarily on manual segmentation, which is labor-intensive and time-consuming, hindering the consistency and efficiency of diagnostic workflows. To date, only a few studies have explored automated segmentation in veterinary imaging. Specifically, deep learning has proven effective for the volumetric analysis of the canine kidneys and adrenal glands, the identification of abnormalities like kidney calculi, and the segmentation of complex structures such as the medial retropharyngeal lymph nodes, demonstrating feasibility in dogs (12–15). Most of these reports employed U-Net or transformer-derived models. Overall, favorable performance was reported for kidney and adrenal gland volumetry and for renal calculi detection, whereas lower performance was observed for medial retropharyngeal lymph-node segmentation, which the authors attributed to the nodes' irregular morphology and their close proximity to surrounding soft tissues, and the limited size of their single-center dataset.

To the best of our knowledge, no previous studies have applied deep learning-based automated segmentation specifically targeting the canine liver, despite its clinical relevance in veterinary medicine. Accurate liver segmentation is beneficial because the precise quantification of liver volume aids in assessing conditions such as hepatomegaly or microhepatica, which are important indicators of underlying diseases (16, 17). Moreover, precise volumetric measurements have proven useful for monitoring postoperative liver regeneration, such as in dogs undergoing surgical correction of portosystemic shunts (PSS) (18–20). Given these clinical implications, efforts have been made in veterinary medicine to optimize liver segmentation procedures through simplified manual methods, such as reducing the total number of CT slices and reorienting the imaging plane (21, 22). Although these approaches partially reduce the workload, they rely on manual segmentation.

Given these challenges, there is a clear need to develop automated and reliable segmentation methods for canine liver CT imaging. Therefore, we aimed to develop a deep learning-based automated segmentation model to reduce the manual effort and time required while maintaining a segmentation accuracy comparable to that of expert manual delineation.

## 2 Materials and methods

### 2.1 Dataset for CT scans

This retrospective study utilized post-contrast CT scans of dogs collected at Hokkaido University Veterinary Teaching Hospital (HUVTH), Japan. Approval from the Animal Care and Use Committee of our institution was not required because of the retrospective nature of the study. A total of 221 CT scans from 206 dogs were included, with some dogs having undergone multiple CT scans for various clinical reasons. All medical records used in this study were fully anonymized. Owing to the limited number of samples, the evaluation was performed at the individual image level rather than at the individual dog level. Therefore, the separation of scans from the same dog across the training, validation, and test sets was not guaranteed. Comprehensive medical records including breed and body weight were reviewed to provide a detailed characterization of the study population.

**Figure 1:**
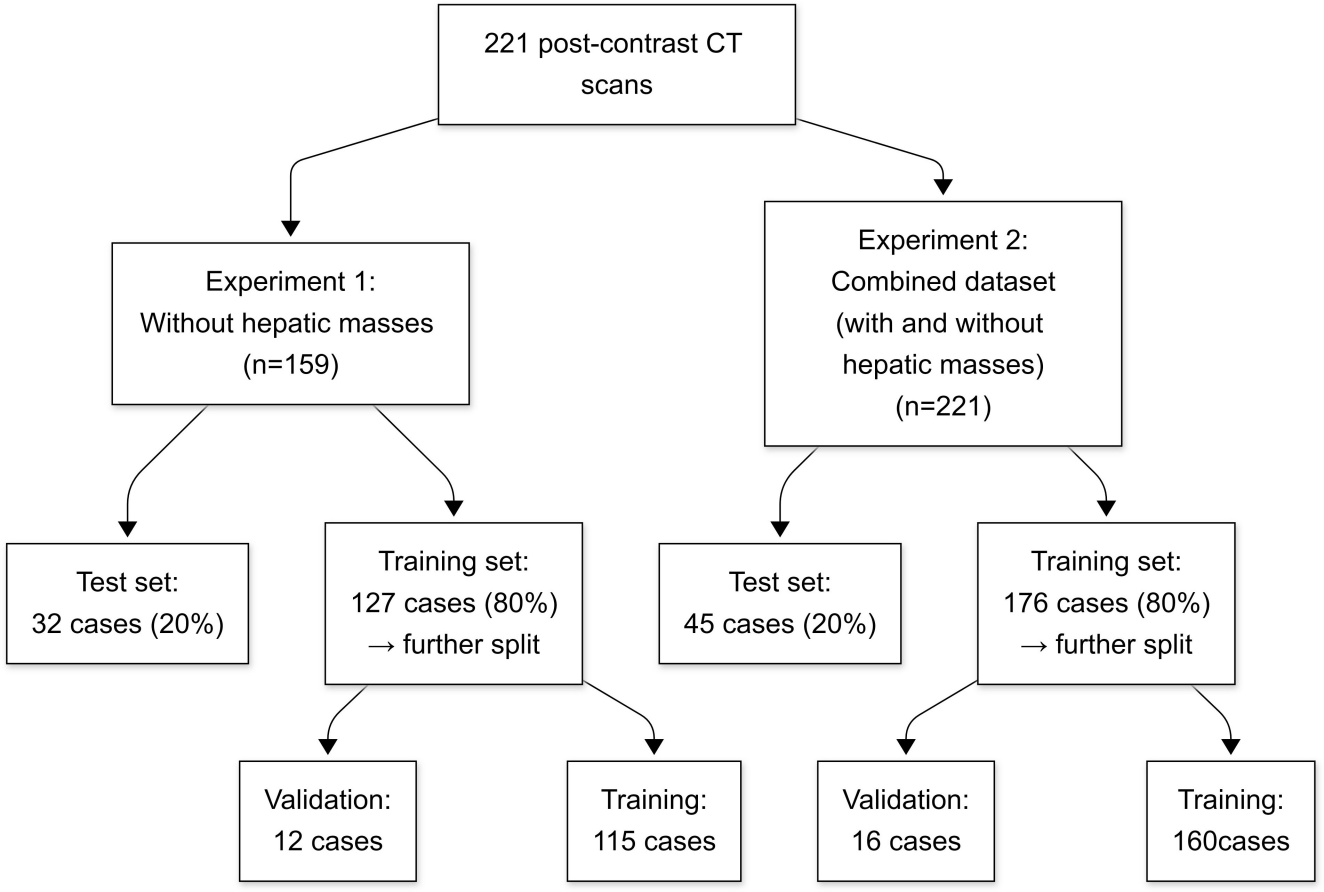
Flowchart of the study design and dataset allocation. Numbers represent the count of CT scans in each group. CT, computed tomography.

The data were structured into two distinct datasets to evaluate the model performance under various liver conditions. The first dataset consisted of 159 CT scans without hepatic masses, obtained from 147 dogs. As an initial step toward automatic liver segmentation, this dataset specifically included scans that demonstrated normal hepatic anatomy. The second dataset combined cases with and without hepatic masses, resulting in 221 CT scans from 206 dogs. This dataset included all the scans from the first dataset and an additional 62 scans from 60 dogs with hepatic masses. Because many abdominal CT scans performed at HUVTH are primarily intended for surgical planning, hepatic masses were included in the second dataset. Cases that could alter hepatic morphology owing to factors other than hepatic masses, such as cirrhosis or PSS, as well as those with a large amount of ascites were excluded because of their limited number, which could adversely affect the robustness of the deep learning model. In the case of PSS, the timing of imaging acquisition at HUVTH differed from the standard imaging protocol used for other abdominal cases, potentially introducing inconsistencies in the training data. Both datasets were randomly shuffled and initially split into training and test sets in an 80:20 ratio. The training set was further subdivided by allocating 10% of the data for validation. For the second dataset, particular attention was paid to ensure a balanced representation of cases with and without masses across the training, validation, and test sets. This stratified splitting aimed to minimize bias during model training and evaluation, thereby providing a robust assessment of the generalizability of the model. The overall study design and the detailed allocation of CT scans into the datasets are summarized in Figure 1.

CT scans were acquired using an 80-row multidetector CT scanner (Aquillion PRIME; Toshiba Medical Systems, Tochigi, Japan) with standardized imaging protocols. The scanning parameters included tube voltage settings of 80–120 kVp, Auto Exposure Control (Sure Exposure 3D, Toshiba Medical Systems, Tochigi, Japan), a 512 × 512 matrix, slice thickness of 0.5 mm, helical pitch of 0.813, and a 0.5-s rotation time. All dogs were anesthetized with propofol induction and maintained using isoflurane inhalation during the CT procedures, positioned in either the dorsal or sternal recumbency position. The contrast agents iohexol (Omnipaque 300, GE Healthcare Co., Ltd., Tokyo, Japan) and iopamidol (Iopamidol 150, Fuji Pharma Co., Ltd., Tokyo, Japan) were administered at a dose of 600 mgI/kg via the cephalic vein using a power injector (Dual Shot, Nemoto Kyorindo Co., Ltd., Tokyo, Japan), with an injection duration of 20 s. Segmentation was conducted using portal venous phase images, although the exact timing of portal venous phase acquisition after contrast injection varied slightly depending on the case.

### 2.2 Manual segmentation

To obtain an accurate and consistent ground truth, a single-annotator segmentation protocol was implemented. A highly skilled veterinary radiology technologist with 15 years of experience performed all manual segmentation on a 3D image analysis workstation (Synapse Vincent, Fujifilm Corporation, Tokyo, Japan). The intrahepatic vessels were included within the operator-defined region of interest. The segmentation results were subsequently reviewed by a veterinary radiology specialist with >10 years of experience (G.S.) to ensure accuracy.

The CT scans were preprocessed to standardize the dataset and ensure compatibility with the deep learning model. All preprocessing steps were implemented using Python, PyTorch and TorchIO library. Binary masks were generated from the manually segmented liver labels by assigning a voxel value of 1 to the liver and 0 to all other areas. This conversion enabled the model to clearly distinguish the liver from the surrounding structures during training. Subsequently, CT images were normalized by scaling their voxel intensities to a range of [−1, 1] using TorchIO's RescaleIntensity transform, thereby enhancing the model's training efficiency. To ensure a uniform input size for the network, all volumes were resized to 256 × 256 × 200 voxels using TorchIO's Resize transform, with linear interpolation applied to the images and nearest-neighbor interpolation to the masks.

### 2.3 Model architecture

**Figure 2:**
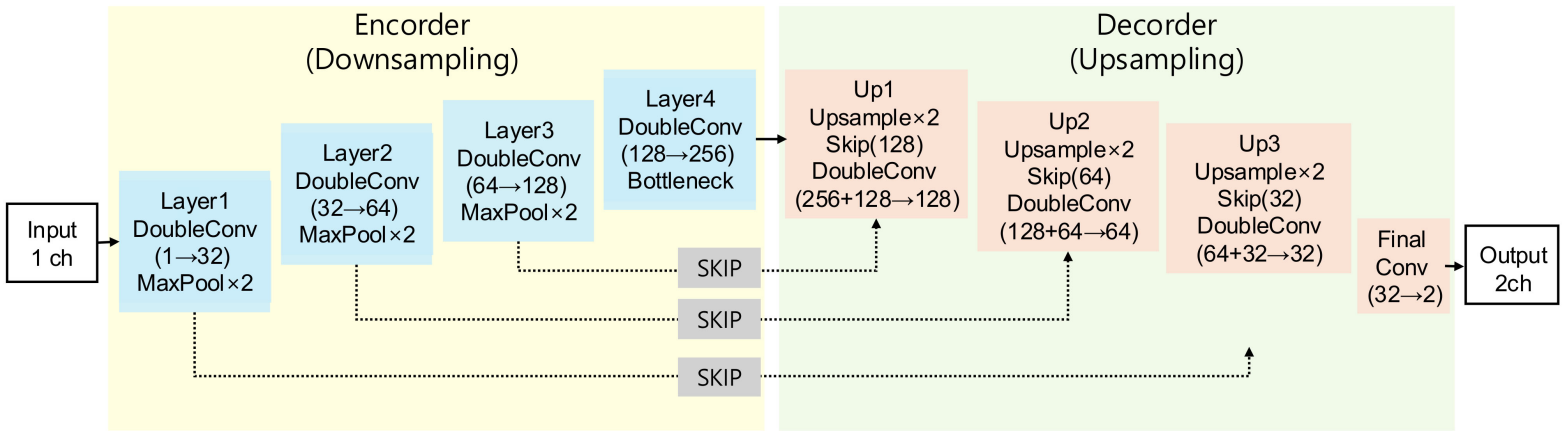
Architecture scheme illustrating the 3D U-Net model used in this study. The model consists of an encoder path **(left)** for feature extraction and a decoder path **(right)** for segmentation map reconstruction, connected by skip connections.

In this study, we employed a three-dimensional (3D) U-Net architecture for liver segmentation of CT images (Figure 2). The network architecture was based on the original U-Net and was modified to support volumetric data by replacing 2D operations with their corresponding 3D counterparts, including 3D convolution, 3D max-pooling, and trilinear upsampling methods (23). The 3D U-Net model uses an encoder-decoder format designed to effectively capture spatial features from 3D CT data. The encoder path includes 3D convolutions and pooling layers to progressively extract higher-level spatial features, whereas the decoder mirrors this structure using transposed convolutions to restore spatial resolution and facilitate precise localization of the liver regions. Skip connections between the encoder and decoder allow the integration of both high- and low-level features, thereby preserving the spatial details and enhancing the segmentation accuracy. These characteristics make 3D U-Net highly suitable for volumetric data and for addressing the anatomical variability of canine livers. In this study, preprocessed CT images and the corresponding binary label masks were used as inputs to the model.

### 2.4 Network parameters

The model was trained for 100 epochs using an NVIDIA RTX 4090 GPU. A stochastic gradient descent optimizer was utilized, configured with a batch size of four, learning rate of 0.01, momentum of 0.9, and weight decay set to 1 × 10^−4^. The learning rate remained constant throughout the training process.

The input data comprised grayscale CT images with a single input channel, and the network produced a two-channel output that represented the background and liver regions. The primary loss function employed was cross-entropy loss, which is a standard and widely adopted choice for multiclass segmentation tasks in medical imaging. To efficiently manage large 3D volumes, the training data were divided into smaller patches of size 96 × 96 × 96 pixels, with each patch overlapping by eight pixels. These patches were systematically extracted using the TorchIO GridSampler, whereas predictions from the model were aggregated into full-volume segmentations during the evaluation using the TorchIO GridAggregator. This patch-based approach optimizes memory usage while maintaining spatial coherence in the segmentation results.

Model performance was monitored during training through periodic evaluations of the validation set. The validation accuracy served as the primary metric for checkpointing, and the top 10 epochs were saved based on this metric to ensure optimal model performance during testing.

### 2.5 Evaluation metrics

To evaluate the model performance, several quantitative metrics were employed to assess the segmentation accuracy between the predicted liver masks and ground truth masks on the test set. The metrics were calculated for each test sample and averaged across the entire dataset to obtain the overall performance indicators. These were computed on the reassembled full-volume predictions aggregated from patch-based evaluations, as described in the Implementation Details section.

The Dice similarity coefficient (DSC) was used to measure the overlap between the predicted and ground truth segmentation masks. This is defined as the ratio of twice the area of intersection to the total area of both the predicted and ground truth masks. The formula used is as follows:


(1) $$\begin{array}{l} DSC=\frac{2\times |P\cap G|}{\left|P|+|G\right|} \end{array}$$

where *P* and *G* denote the predicted and ground truth masks, respectively.

Sensitivity was calculated to evaluate the ability of the model to correctly identify the positive pixels in the segmentation mask. This is defined as the ratio of true-positive pixels to the sum of true-positive and false-negative pixels:


(2) $$\text{Sensitivity=}\frac{\text{True Positive}}{\text{True Positive + False Negative}}$$

Specificity measures the ability of the model to correctly identify negative pixels (background) and is calculated as the ratio of true-negative pixels to the sum of true-negative and false-positive pixels:


(3) $$\text{Specificity = }\frac{\text{True Negative}}{\text{True Negative + False Positive}}$$

Precision quantifies the proportion of true-positive pixels among all pixels classified as positive by the model:


(4) $$\text{Precision = }\frac{\text{True Positive}}{\text{True Positive + False Positive}}$$

The overall accuracy of the model was calculated as the ratio of correctly classified pixels (positive and negative) to the total number of pixels in the segmentation mask:


(5) $$\text{Accuracy = }\frac{\text{True Positive + True Negative}}{\text{Total Pixels}}$$

The intersection-over-union (IoU), also known as the Jaccard Index, measures the overlap between the predicted and ground truth masks by dividing the intersection area by the union area:


(6) $$\text{IoU}=\frac{\left|P\cap G\right|}{\left|P\cup G\right|}$$

The volume ratio was computed to assess the volumetric accuracy of the segmentation. This is defined as the ratio of the predicted liver volume to the ground truth liver volume. Each liver volume was calculated by multiplying the number of positive voxels in the respective segmentation mask by the voxel volume derived from the original CT scan as follows:


(7) $$\text{Volume ratio =}\frac{\text{Number of positive Voxels in Predicted Mask}\times \text{Voxel volume}}{\text{Number of positive Voxels in Ground Truth Mask}\times \text{Voxel volume}}$$

This patch-based evaluation approach, combined with metrics computed from aggregated full-volume predictions, ensured consistency and reliability in assessing segmentation accuracy. All metrics were implemented using Python and PyTorch and calculated voxel-wise across the entire 3D volume. In addition, Pearson correlation coefficient (r) was calculated to assess the linear relationship between the predicted and manually measured liver volumes.

## 3 Results

### 3.1 Evaluation of liver segmentation on the dataset without hepatic masses (Experiment 1)

**Figure 3:**
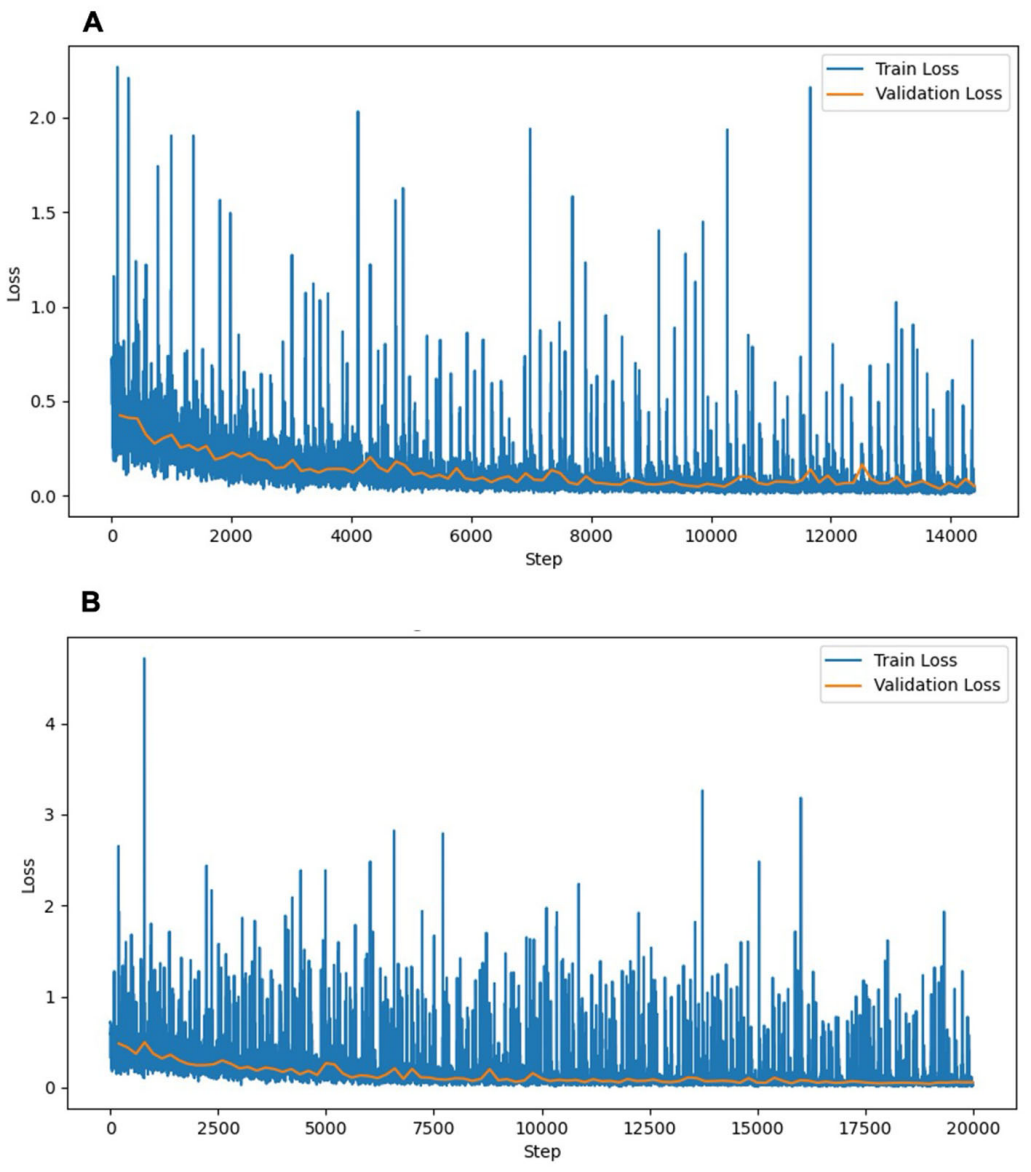
Training and validation loss curves of the liver segmentation models. **(A)** Experiment 1 (without hepatic masses). **(B)** Experiment 2 (combined dataset with and without hepatic masses). The training loss (blue) and validation loss (orange) decreased steadily over the training steps, indicating stable convergence without notable overfitting. The training loss curve in Experiment 2 exhibited higher fluctuations, reflecting the increased complexity associated with the diverse morphological features of hepatic masses. The validation loss curves remained similarly stable across both experiments, indicating consistent generalization.

**Figure 4:**
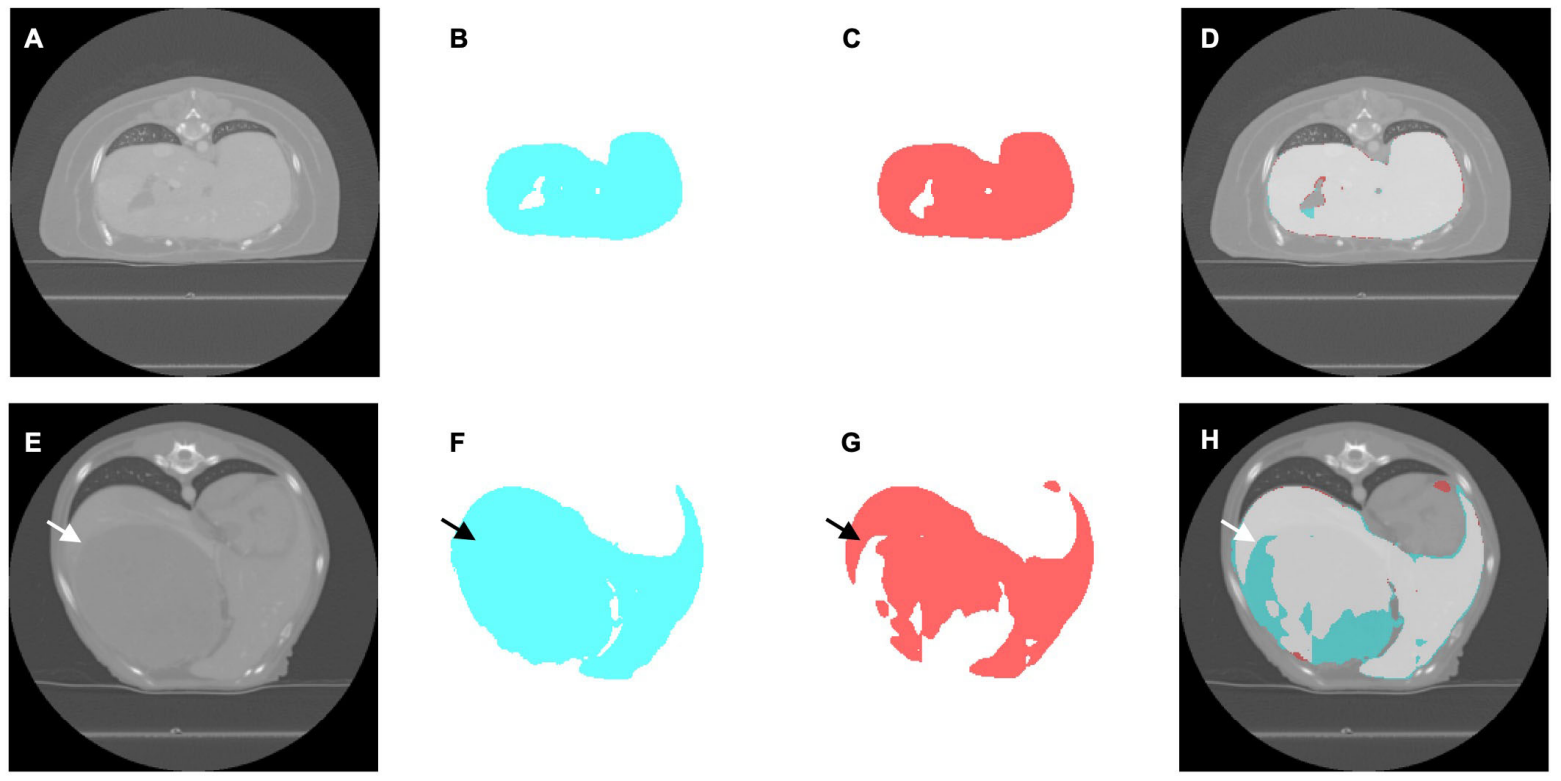
Representative computed tomography (CT) slices illustrating liver segmentation in cases without **(A–D)** and with **(E–H)** hepatic masses. **(A, E)** Original CT image. **(B, F)** Ground truth segmentation masks generated by expert radiologists (cyan). **(C, G)** Segmentation masks predicted using the deep learning model (red). **(D, H)** Overlay images demonstrating segmentation accuracy, indicating overlapping regions between the ground truth and model predictions (light gray), regions segmented only by experts (cyan), and regions segmented only by the model (red). The arrows indicate the location of the hepatic mass.

This experiment used a dataset of 159 CT scans without hepatic masses, divided into 115, 12, and 32 scans for training, validation, and testing, respectively. Table 1 provides an overview of these scans, including the number of dogs, median body weight, and median liver volume. Regarding the training performance, the model showed stable convergence, with mean training and validation losses of 0.124 and 0.125, respectively (Figure 3A). For the test set, the model achieved an accuracy of 0.981 and a loss of 0.05. The detailed segmentation metrics are summarized in Table 2, revealing a mean DSC of 0.926, IoU of 0.865, and volume ratio of 1.042, indicating strong volumetric agreement between the predicted and ground truth masks. The additional metrics included sensitivity (0.946), specificity (0.995), precision (0.910), and accuracy (0.992). Figures 4A–D illustrates a representative normal liver slice and its segmentation overlay, demonstrating the precise delineation of the liver boundaries.

**Table 1:** Characteristics of the study datasets, including breed distribution, median body weight, and median liver volume.

**Group**	**Number of cases**	**Median body weight (kg) [IQR]**	**Median liver volume (mL) [IQR]**	**Breeds included (number of dogs)**
Without hepatic masses	159	9.05 [6–18]	297 [192–511]	Mixed (22), Miniature Dachshund (21), Shiba (12), Toy Poodle (12), Labrador Retriever (9), French Bulldog (8), Golden Retriever (8), Miniature Schnauzer (6), Pomeranian (6), Jack Russell Terrier (5), Shetland Sheepdog (5), Bernese Mountain Dog (4), Border Collie (4), Bulldog (4), Chihuahua (4), Samoyed (4), Shih Tzu (4), Welsh Corgi (4), American Cocker Spaniel (2), Bichon Frise (2), Hokkaido (2), Italian Greyhound (2), Pekingese (2), Boston Terrier (1), Cavalier King Charles Spaniel (1), English Cocker Spaniel (1), Flat-coated Retriever (1), Miniature Pinscher (1), Scottish Terrier (1), Standard Poodle (1)
With hepatic masses	62	7.55 [5.03–10.95]	427.5 [245.5–721.5]	Miniature Dachshund (11), Mixed (11), Toy Poodle (9), Shiba (4), Shih Tzu (4), Border Collie (3), Samoyed (3), Yorkshire Terrier (3), Miniature Schnauzer (2), Welsh Corgi (2), Chihuahua (1), French Bulldog (1), Jack Russell Terrier (1), Labrador Retriever (1), Papillon (1), Scottish Terrier (1), Shetland Sheepdog (1), Standard Poodle (1), West Highland White Terrier (1), Wire Fox Terrier (1)
Overall total	221	8.4 [5.53–14.88]	318 [204–563]	Mixed (33), Miniature Dachshund (32), Toy Poodle (21), Shiba (16), Labrador Retriever (10), French Bulldog (9), Miniature Schnauzer (8), Shih Tzu (8), Golden Retriever (8), Border Collie (7), Samoyed (7), Jack Russell Terrier (6), Pomeranian (6), Shetland Sheepdog (6), Welsh Corgi (6), Chihuahua (5), Bernese Mountain Dog (4), Bulldog (4), Yorkshire Terrier (3), American Cocker Spaniel (2), Bichon Frise (2), Hokkaido (2), Italian Greyhound (2), Pekingese (2), Scottish Terrier (2), Standard Poodle (2), Boston Terrier (1), Cavalier King Charles Spaniel (1), English Cocker Spaniel (1), Flat-coated Retriever (1), Miniature Pinscher (1), Papillon (1), West Highland White Terrier (1), Wire Fox Terrier (1)

Values are presented as median [IQR]. IQR, Interquartile range.

**Table 2:** Quantitative segmentation performance metrics of the two experiments on the test set.

**Experiment**	**DSC**	**IoU**	**Sensitivity**	**Specificity**	**Precision**	**Accuracy**	**Volume ratio**
Exp 1: Without hepatic masses	0.926	0.865	0.946	0.995	0.910	0.992	1.042
Exp 2: Combined dataset	0.929	0.868	0.931	0.995	0.928	0.991	1.006
Without hepatic masses subset	0.931	0.872	0.942	0.995	0.921	0.992	1.025
With hepatic masses subset	0.924	0.861	0.906	0.994	0.945	0.987	0.959

DSC, dice similarity coefficient; IoU, intersection-over-union.

### 3.2 Evaluation of liver segmentation on the combined dataset of cases with and without hepatic masses (Experiment 2)

In this experiment, the original 159 CT scans without hepatic masses were supplemented with 62 cases exhibiting hepatic masses, resulting in 221 CT scans, which were divided into 160 for training, 16 for validation, and 45 for testing. Table 1 summarizes the characteristics of both datasets, including the breed distribution, median body weight, and median liver volume. The model trained on this more heterogeneous dataset exhibited a mean training loss of 0.170 and validation loss of 0.132 (Figure 3B). For the test set, the model achieved an accuracy of 0.980 and a loss of 0.057. Table 2 shows a mean DSC of 0.929, an IoU of 0.868, and volume ratio of 1.006, which is slightly higher than that in Experiment 1. Sensitivity (0.931), specificity (0.995), precision (0.928), and accuracy (0.991) remained high. However, a subset analysis of the test set, which involved dividing it into cases with and without hepatic masses, revealed that the segmentation performance was higher for cases without hepatic masses (DSC = 0.931) than for those with hepatic masses (DSC = 0.924). Figures 4E–H shows a sample slice from the liver with a hepatic mass, demonstrating greater morphological variability owing to the presence of the hepatic mass.

### 3.3 Correlation analysis between predicted and manually measured liver volumes

**Figure 5:**
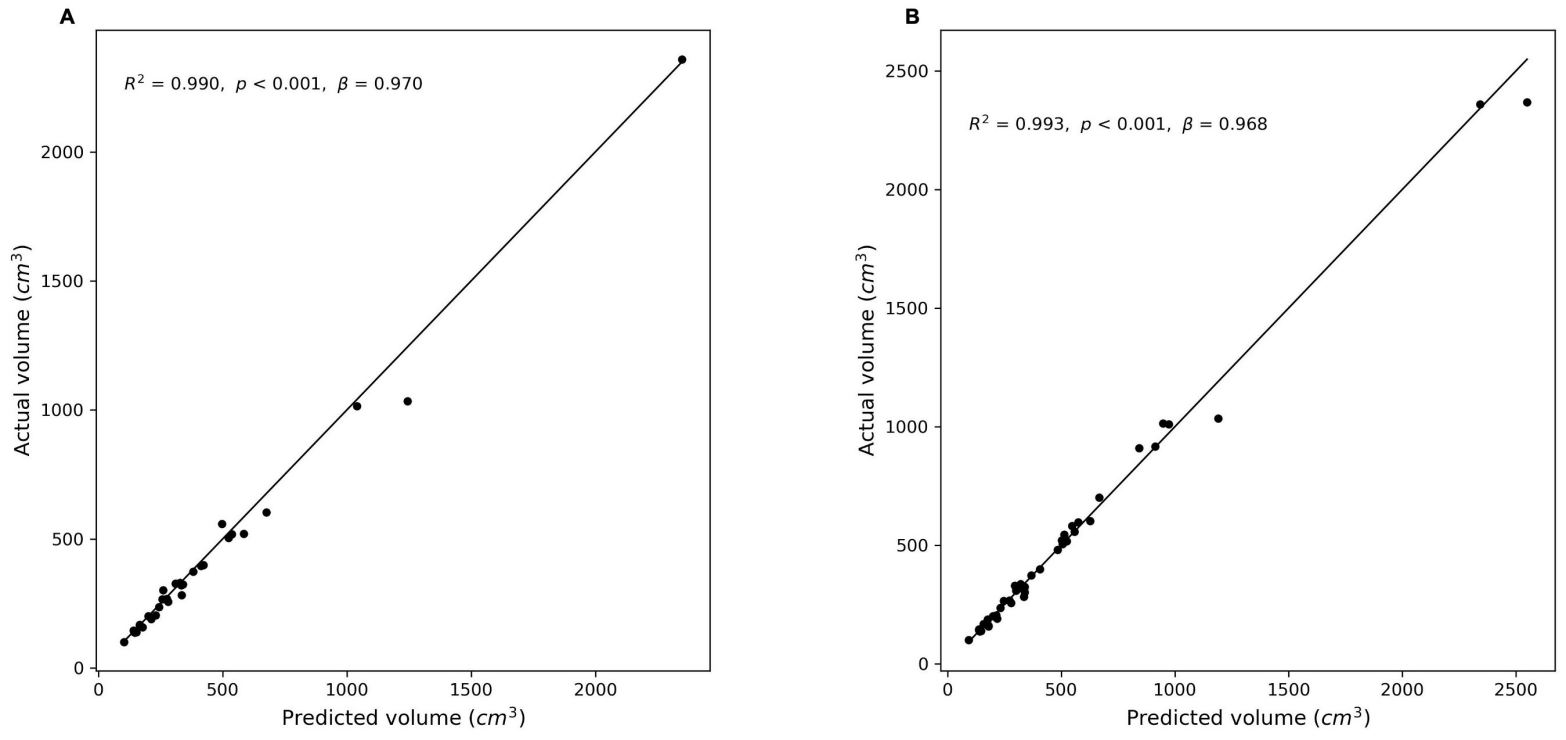
Correlation between predicted and manually measured liver volumes. **(A)** Experiment 1 (without hepatic masses). **(B)** Experiment 2 (combined dataset with and without hepatic masses). Scatter plots illustrate the relationship between the actual (manually segmented) liver volumes and the volumes predicted by the segmentation models. The diagonal line (*y* = *x*) represents perfect prediction. **(A)**: *r* = 0.995, *R*^2^ = 0.990, *p* < 0.001; **(B)**: *r* = 0.997, *R*^2^ = 0.993, *p* < 0.001).

Figure 5 illustrates the correlation between the manually measured and predicted liver volumes in both experiments. Experiment 1 showed a strong correlation (*r* = 0.995, *R*^2^ = 0.990), and Experiment 2, which was conducted using a combined dataset of cases with and without hepatic masses, demonstrated slightly higher agreement (*r* = 0.997, *R*^2^ = 0.993). Most predictions were closely aligned with the ideal correlation line, although slightly increased variability was observed in Experiment 2.

## 4 Discussion

This study is the first to develop a deep learning-based model specifically for canine liver segmentation in CT images, addressing the existing gap in veterinary medicine. The model trained exclusively on cases without hepatic masses (Experiment 1) demonstrated strong performance, with a mean DSC of 0.926 and an IoU of 0.865 (Table 2). These results indicate that the model reliably segments the liver in cases without significant anatomical complexities such as hepatic masses. Establishing such a performance baseline is essential because it provides a clear reference for interpreting segmentation performance in anatomically more complex cases involving hepatic masses. This baseline helps clarify whether decreased segmentation performance results primarily from anatomical variations caused by lesions, leading to distributional shifts, or from the intrinsic limitations of the segmentation model itself. A previous study highlighted the influence of distributional shifts on model performance when encountering previously unseen anatomical characteristics (24). Thus, the results of Experiment 1 serve as a robust benchmark for subsequent evaluations involving more diverse and complex hepatic conditions.

In contrast, the model trained on a combined dataset, which included cases with and without hepatic masses (Experiment 2), achieved a slightly higher segmentation performance, with a DSC of 0.929 and an IoU of 0.868 (Table 2), suggesting that the increased dataset size and diversity may contribute to improved model robustness. Human medical research has previously demonstrated that training on diverse datasets covering a broad age range and multiple abnormalities such as tumors, inflammation, and vascular disorders can enhance model generalizability (25). Similarly, our study showed improved general segmentation performance when the training included cases with and without hepatic masses. Nevertheless, the model exhibited increased segmentation variability and a slight decrease in performance in cases involving hepatic masses, likely because of the limited number of such cases in our dataset. This limitation may have restricted the ability of the model to fully learn the morphological variability associated with hepatic masses.

Further insights were gained through the analysis of the volume ratio metric, which is defined as the ratio between the predicted liver volume and the volume derived from the ground truth masks. This metric demonstrated a strong agreement between the predicted and ground truth volumes. However, because it does not directly assess spatial accuracy, it should be complemented with spatially sensitive methods. Relying solely on the volume ratio may overlook spatial discrepancies caused by complex or irregular morphologies. Thus, it is important to use a combination of different evaluation metrics to assess distinct aspects and provide complementary information (26). Spatially sensitive metrics such as DSC and IoU, which evaluate spatial overlap, are valuable for assessing anatomically irregular cases (27). In this study, we comprehensively evaluated segmentation performance by incorporating multiple metrics.

The performance of the model closely aligns with that of the leading liver segmentation methods used in medical imaging. For instance, top-ranking algorithms in the Liver Tumor Segmentation Challenge consistently report DSCs between 0.93 and 0.96, which is comparable to expert manual segmentation (28). This comparison highlights the effectiveness of our approach, particularly given the inherent challenges in veterinary imaging, such as significant inter-breed variations in liver shape and size, and relatively limited sample sizes compared to human studies. Considering these challenges, achieving DSC and IoU values that closely match those of human studies further demonstrates that advanced segmentation techniques can be effectively adapted to veterinary imaging, despite these dataset limitations.

Veterinary imaging presents unique complexities distinct from those in human medicine, primarily due to breed-related anatomical variations and diverse clinical presentations. Similar challenges have been reported in canine kidney segmentation studies (12). Such factors complicate the segmentation task, particularly in livers with hepatic masses, which typically exhibit greater morphological variability than human livers, as demonstrated by the decreased segmentation performance and increased segmentation variability observed in Experiment 2. Furthermore, although the dataset in this study, consisting of 221 CT scans from 206 dogs, is large compared with several previous veterinary imaging studies, such as those involving 40 (14) and 76 dogs (13), it remains relatively modest compared with human imaging databases (29). Nonetheless, the current findings clearly demonstrate that deep learning-based segmentation models can effectively overcome these complexities, achieve competitive performance. This highlights their clinical applicability, in providing rapid and accurate liver volumetry for diagnosing conditions like hepatomegaly or microhepatica, aiding pre-operative planning for mass resection, and enabling objective monitoring of postoperative liver regeneration in veterinary medicine.

This study has some limitations. First, the dataset size was derived from a single institution and was small compared with studies in human medicine, with a limited representation of various breeds and clinical conditions. A larger and more diverse dataset could enhance the robustness and adaptability of the model. Although some studies in human medicine have demonstrated good segmentation performance using comparable or smaller datasets, the inherent variability in veterinary medicine owing to breed differences suggests that similar-sized datasets may not sufficiently represent this diversity (28, 30). The model's generalizability could be further enhanced by employing data augmentation to compensate for the limited dataset size. The evaluation in this study was performed at the image level rather than at the individual dog level, without ensuring the separation of dogs across the training, validation, and test sets, potentially influencing model generalizability. Additionally, although various dog sizes, ranging from small to large breeds, were included, the breed size distribution across subsets was not controlled, further limiting the representativeness of our dataset.

To address these limitations, several future directions are planned. Multi-institutional validation is essential to assess the model's robustness across diverse imaging conditions. This issue is particularly relevant in veterinary medicine, where there is no clear scientific consensus regarding optimal patient positioning, leading to substantial variations among institutions (31). Transfer learning could also be explored, using models pre-trained on large human liver datasets (32). Finally, future work could explore the use of other loss functions, such as focal loss, to potentially enhance segmentation accuracy, particularly for challenging cases involving hepatic masses.

## 5 Conclusion

This study presents the first deep learning-based liver segmentation model specifically developed for veterinary medicine to achieve reliable segmentation results. Including hepatic masses improved the generalizability but slightly increased the segmentation variability, whereas simpler anatomies without lesions resulted in a more consistent performance. Despite challenges such as breed-specific anatomical variations, limited sample sizes, and non-standardized imaging protocols, this approach demonstrates considerable potential for enhancing diagnostic precision, reducing clinician workload, and expediting decision-making in veterinary practice. Future research should prioritize expanding the dataset to include a broader variety of clinical cases, performing external validations, and exploring hybrid training strategies to further enhance the reliability and clinical applicability of the model.

## Data Availability

The raw data supporting the conclusions of this article will be made available by the authors, without undue reservation.
